# #IStandWithDan versus #DictatorDan: the polarised dynamics of Twitter discussions about Victoria’s COVID-19 restrictions

**DOI:** 10.1177/1329878X20981780

**Published:** 2021-05

**Authors:** Timothy Graham, Axel Bruns, Daniel Angus, Edward Hurcombe, Sam Hames

**Affiliations:** Queensland University of Technology, Australia

**Keywords:** clicktivism, Coronavirus, COVID-19, disinformation, hashtag activism, misinformation, multi-step flow model, social media, Twitter

## Abstract

In this article, we examine two interrelated hashtag campaigns that formed in response to the Victorian State Government’s handling of Australia’s most significant COVID-19 second wave of mid-to-late 2020. Through a mixed-methods approach that includes descriptive statistical analysis, qualitative content analysis, network analysis, computational sentiment analysis and social bot detection, we reveal how a small number of hyper-partisan pro- and anti-government campaigners were able to mobilise *ad hoc* communities on Twitter, and – in the case of the anti-government hashtag campaign – co-opt journalists and politicians through a multi-step flow process to amplify their message. Our comprehensive analysis of Twitter data from these campaigns offers insights into the evolution of political hashtag campaigns, how actors involved in these specific campaigns were able to exploit specific dynamics of Twitter and the broader media and political establishment to progress their hyper-partisan agendas, and the utility of mixed-method approaches in helping render the dynamics of such campaigns visible.

## Introduction

As the coronavirus pandemic continues, discussions about appropriate public policy aimed at its management and mitigation have intensified. Even in regions that have seen a comparatively high political and societal consensus about the need for severe lockdowns and other interventions aimed at arresting the spread of the virus, such unity is gradually coming unstuck. Coordinated by the ‘national cabinet’ that included the Prime Minister as well as state and territory premiers and chief ministers, for example, Australian governments were comparatively unanimous in their initial responses to the pandemic, and party-political squabbles between leaders of different ideological hues seemed temporarily suspended at least on these measures; over time, however, as infection dynamics developed differently across the various states and territories, unity disintegrated, and by late-2020, there were open recriminations between state leaders, and between states and the Prime Minister, over the interstate border closures and local lockdown measures introduced in different Australian regions.

Such acrimony has been most heated in the context of the lockdowns and border closures instituted in Victoria, Australia’s second most populous state, which saw the greatest number of COVID-19 infections and deaths and, in particular, experienced a substantial second wave of infections from mid-June 2020 onwards (Victorian State Government, 2020) that was managed by increasingly severe lockdowns. This second major outbreak generated substantial and controversial debate in the media and within the general population, centring both on the concrete reasons for the new outbreak, and on the appropriate level of lockdown restrictions and the roadmap towards reducing them again as the new outbreak subsided.

Much of the criticism of these measures focussed on the Victorian Premier, Daniel Andrews of the Australian Labor Party, who had become the public face of the state’s response to the coronavirus pandemic not least through an uninterrupted series of (at the time of writing) more than 100 daily press conferences on his government’s actions. Although enjoying high public approval ratings in Victoria through most of 2020, Andrews was attacked increasingly harshly by his political opponents in state and federal politics; scrutinised critically by state and federal news media; and in mid-October 2020 his electorate office was vandalised by unknown assailants (Sakkal and Towell, 2020).

Claims of growing public frustration with the Victorian government’s measures, as reported in state and national media, were also exploited by the state opposition, led by parliamentary Opposition Leader Michael O’Brien from the Liberal Party. Its attacks focussed especially on two perceived faults with government policy: on one hand, they highlighted the impact of lockdown restrictions on the Victorian economy, and advocated for a more accelerated re-opening of local businesses in spite of a low level of continuing community transmission of the COVID-19 virus (ABC News, 2020); on the other hand, they pointed to failures in the management of the mandatory hotel quarantine for travellers returning to Victoria, where the use of poorly trained private security guards resulted in quarantine breaches, and the inadequate response to outbreaks in aged-care homes, where the majority of COVID-related deaths occurred. Overall, the opposition blamed the government, in general, and Premier Andrews, in particular, for the infections and deaths that ensued especially in the second wave (Fowler and Ilanbey, 2020).

**Figure 1.**
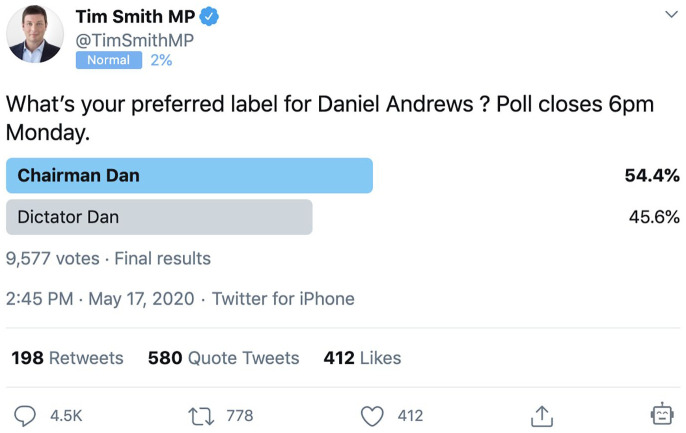
Twitter poll by @TimSmithMP, which greatly amplified the visibility of the #DictatorDan epithet and encouraged take-up of the hashtag on Twitter.

The most aggressive opposition spokesperson pursuing this line of attack was the Liberal MP for the electorate of Kew, Tim Smith. In a series of media appearances, particularly on breakfast news programmes, as well as in his social media posts (see, for example, Figure 1), he sought to establish a number of negative epithets for Andrews, including ‘Chairman Dan’ (implying that the Labor Premier was running the state in the style of an oppressive communist regime) and even ‘Dictator Dan’ (Willingham, 2020). Such attacks on Andrews, presented as simple and memorable slogans, were clearly calculated also as attempts to generate broader take-up in public discussions of the government’s measures against the pandemic, not least on social media; indeed, Smith’s own social media posts also sought to promote Twitter hashtags such as #ChairmanDan and #DictatorDan. Subsequent criticism of the Victorian pandemic response, by Smith and others, also gave rise to the Twitter hashtag #DanLiedPeopleDied, as well as resulting in the #IStandWithDan hashtag expressing opposition to such attacks and support for the Premier.

In this article, we examine this take-up of attacks on Andrews within social media debate, as well as the emergence of responses that counter such attacks. We focus here especially on Twitter – a platform that has been shown to be a particularly important space for political discussion in Australia (Bruns and Burgess, 2015; Sauter and Bruns, 2015).

Such take-up could be regarded *prima facie* as evidence of a two-step flow (Katz, 1957), from political opinion leaders to the general public, demonstrating the continued relevance of communication theories from the pre-digital era even in a thoroughly mediatised present where social media logics exert increasing influence over public and political debate (Van Dijck and Poell, 2013). Closer investigation, however, reveals a considerably more complex flow of ideas across multiple steps (cf. Ognyanova and Monge, 2013): not only is it possible that MP Smith and others are not themselves the originators of these attacks against Premier Andrews, but merely amplify lines of attack that were developed by party strategists or other groups seeking to undermine Andrews (i.e. that there is a preceding step in the information flow from these groups *to* Smith and colleagues); but we also find evidence that the broader adoption and dissemination of language targeting Andrews is driven at least in part by coordinated and apparently inauthentic activity that amplifies the visibility of such language before it is adopted by genuine Twitter users.

This would represent a further step in the information flow, from Smith and other Andrews opponents via such coordinated, artificial amplification to the general Twitter public – from where, in a further step in the flow of information, it is then also picked up by journalists and opinion writers, and transported into additional media reporting. Our study, then, presents the evidence for this multi-step, deliberately manipulated flow of information, and compares it with our observations of the response to these attacks.

## Data collection and methods

The dataset for this study was collected using the Twarc open source library (DocNow/twarc, 2020) through the Twitter Enterprise application programming interface (API). Twarc is a command line tool for collecting and archiving tweets. This process involves specifying a hashtag and/or search term of interest, for example, ‘#IStandWithDan’, and twarc returns tweets within a 7-day window containing the search query. The 7-day window is too restrictive for our analysis, therefore, we purchased access to the Twitter Enterprise API to enable historical tweet collection. The collection contains all tweets from 1 March 2020 to 25 September 2020 containing any of the following hashtags: #IStandWithDan, #DictatorDan or #DanLiedPeopleDied.^1^ We chose this timeframe to fully cover the onset of the second wave of the coronavirus outbreak in Victoria, including the months immediately preceding it when national restrictions were already in place. These three hashtags were purposively selected as a basis for investigating Twitter discussions both *against* (#DictatorDan and #DanLiedPeopleDied) and *in support of* the Victorian Premier (#IStandWithDan). All three hashtags featured regularly on Twitter’s list of Australian trending topics, and attracted considerable scholarly (Graham, 2020) and media (Media Watch, 2020) attention. The resulting dataset contains 396,983 tweets sent by 40,203 accounts, indicating substantial repeat usage of these hashtags by many participating accounts.

We use a mixed-methods approach for data analysis, including descriptive statistical analysis; in-depth close reading and qualitative content analysis of tweets and account profiles; network analysis; sentiment analysis; and social bot detection using machine learning. For the network analysis, we construct a hashtag network, where Twitter accounts and hashtags are nodes, and links between nodes represent the number of times that account A used hashtag B in a tweet. This type of network affords a socio-semantic analysis of the relationality between accounts and hashtags, revealing the structure of the hashtag publics (Bruns and Burgess, 2015) emerging around the three main hashtags in this study.

Second, we examine the interaction patterns, with particular focus on the most active Twitter accounts, and those receiving the greatest number of @mentions and retweets. This analysis provides an overall perspective of the visibility of, and engagement with, actors in the information space spanned by the hashtags.

Next, we undertake qualitative content analysis of the top 50 most active accounts (by tweet frequency) posting each of the three hashtags. Given that the top 50 accounts represent a considerable proportion of the tweet volume for each hashtag, the purpose of this analysis is to examine whether these 150 accounts appear to represent real, authentic users, or are anonymous accounts that feature a constructed profile, often known as ‘sockpuppets’ (Kumar et al., 2017). Sockpuppet accounts are a long-standing phenomenon on websites and platforms that afford anonymity (Stone and Richtel, 2007). On Twitter, sockpuppets typically present as anonymous accounts, often with fabricated profiles using images taken from the web, that distort and manipulate public opinion by showing support and/or opposition to products, people, or events (Crabb et al., 2015). To undertake analysis of highly active sockpuppet accounts in our dataset, we developed a binary schema to deductively code each account into two categories: ‘authentic’ and ‘sockpuppet’. The process involved a qualitative close reading of each of the 150 accounts to classify it as one category or the other, with the classification verified independently by two of the authors. The codebook operationalises each category as follows.

An *authentic* account is defined as an account with sufficient evidence of being a real person or entity, including a profile photo that is not a stock image or stolen from the web (i.e. reverse image search engines show zero results aside from the account itself); tweets that explicitly or implicitly include personal details and/or post original photos that do not appear elsewhere on the web; and a tweet history that covers a range of topics, even if there are periods of sustained interest in one or two particular topics for a given time period.

However, a *sockpuppet* account is defined as an account with anonymous and/or clearly fabricated profile details, where the actor(s) controlling the account are not identifiable. Sockpuppet accounts exhibit a range of features: no profile photo or a picture stolen from the web (i.e. a reverse image search shows multiple results from different sources); a profile that provides little or no biographical information; an account that was set up recently and/or shows evidence of being set up in haste (e.g. not changing the default Twitter account name, which ends in a sequence of numbers); a tweet history that shows the account only focuses on one or two topics and rarely posts about anything else; and/or a mismatch between the displayed ‘real’ name and the account name.

We note that, in a small number of cases where an account did not clearly belong to one or the other category, we have erred on the side of caution and labelled it as authentic.

Furthermore, we examine the presence of ‘social bots’, or computer-controlled Twitter accounts, across the three hashtags. We use the state-of-the-art Botometer bot detection tool (Sayyadiharikandeh et al, 2020), which uses a machine learning-based approach to score a given Twitter account based on how likely it is to be fully automated. Specifically, we focus on the Completely Automated Probability (CAP) metric, a score between 0 and 1 that defines the probability that an account with this score or greater is controlled by software, that is, *is* a bot in the literal sense of the term (Sayyadiharikandeh et al., 2020). Due to rate limits with both the Botometer tool and the Twitter API, we focus this analysis on a sample of the 1000 most active accounts for each hashtag (by number of tweets), covering a total of 3000 accounts. Although this limits the generalisability of our findings, it focuses on the group of accounts that contributed by far the most activity to each hashtag, and provides a useful assessment of whether, and to what extent, there is evidence of bot activity in these discussions, and how this varies between the hashtags.

Finally, we analyse the emotional valence of the tweets relating to each of the three hashtags under examination. For this, we employ a computational tool known as VADER: Valence Aware Dictionary and sEntiment Reasoner (Hutto and Gilbert, 2014). VADER is a lexicon- and rule-based tool that quantifies sentiment in textual social media data. It has been empirically validated by multiple human judges and obtains human-level accuracy in calculating the sentiment of texts in microblog contexts, including tweets. VADER’s lexicon ratings for individual words range from −4 to +4, with 0 representing ‘neutral’; for any given text of multiple words, the ‘compound score’ metrics provides a sum of all the lexicon ratings of words in the text, normalised to a value between −1 (very negative) and +1 (very positive). For this study, we focus on the compound sentiment score at the level of each tweet. Although VADER and similar tools are by no means perfect, at scale they provide a useful heuristic for understanding the discourses surrounding particular hashtags and/or clusters of activity.

## Analysis

### Hashtag publics

In the first place, we find that #IStandWithDan received considerably more tweet volume than the two ‘anti-Dan’ hashtags. #IStandWithDan attracted 275,573 tweets, or roughly 2.5 times as many as #DictatorDan (107,784 tweets), and 13 times the number of #DanLiedPeopleDied (20,793 tweets). The volume of unique accounts posting these hashtags tells a slightly different story: 27,255 unique accounts posted to #IStandWithDan, 18,030 unique accounts posted to #DictatorDan and 5555 unique accounts posted to #DanLiedPeopleDied.

**Figure 2.**
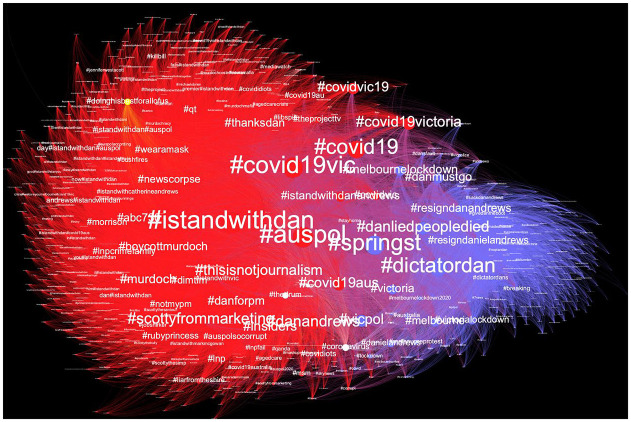
Two-mode hashtag network derived from the complete dataset. Nodes are accounts and hashtags, edges are weighted by number of times user A posted hashtag B, colours represent community clusters (modularity), node size is proportional to in-degree, network is filtered by minimum degree = 50.

Figure 2 shows a network visualisation of the account-to-hashtag relations, clearly illustrating two polarised yet interconnected *ad hoc* publics (Bruns and Burgess, 2015) that form around the pro- and anti-Dan hashtags. The nodes and edges in Figure 1 are coloured by community cluster using the Louvain modularity algorithm (Blondel et al., 2008): the large red cluster centres on the #IStandWithDan hashtag, and the smaller blue cluster on the #DictatorDan and #DanLiedPeopleDied, which are closely interrelated. The other hashtags in the graph appear because they were used in the same tweet alongside one or more of these three core hashtags; unsurprisingly, they often represent other pro-Andrews and pro-Labor hashtags in the #IStandWithDan cluster, and anti-Andrews and pro-Liberal hashtags in the #DictatorDan and #DanLiedPeopleDied cluster.

But we also note that as a result of this use of additional hashtags, the two pro- and anti-Dan clusters are not entirely polarised and disconnected: they are linked by generic hashtags including #auspol, #SpringSt (a common hashtag for state political discussions, in reference to the location of the Victorian parliament), #covid19 and #covid19vic. These hashtags organise general discussions about Australian and Victorian politics and the national and state coronavirus outbreak, and are used in similar ways by participants of all political persuasions; in doing so, they enable followers of these generic hashtags to encounter tweets with both pro- and anti-Andrews hashtags and views.

Partly as a result, then, the connective tissue between the two polarised clusters also comprises accounts that engage with the hashtags they oppose: this is particularly the case for #IStandWithDan, which accounts opposing Daniel Andrews attempt to hijack in a critical and divisive manner at various times. Such oppositional participation may be regarded as an attempt to establish a counterpublic presence within these hashtags; a simpler explanation, however, is that participants merely aimed to disrupt these hashtags altogether, and to discourage their opponents from continued use. In each case, this proved unsuccessful, however.

### The origins and dynamics of the #DictatorDan and #DanLiedPeopleDied hashtags

**Figure 3.**
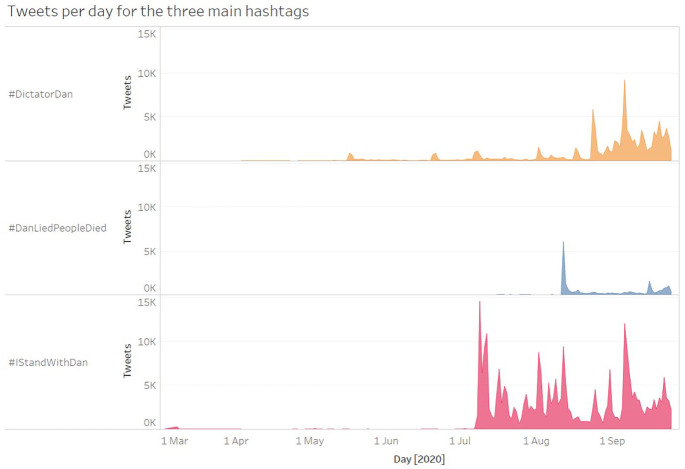
Volume of tweets per day, distinguished by hashtag.

Turning our attention to the broad temporal patterns of the three hashtags, Figure 3 shows their respective volume of tweets per day, from 1 March to 25 September 2020. There is little tweeting activity for any of the hashtags up to 17 May, at which point we observe a spike of over 800 #DictatorDan tweets in 1 day, declining to a small but sustained volume of 100–200 tweets per day in subsequent weeks. On 9 and 10 July, #IStandWithDan use grows substantially, with over 16,000 tweets during its first 2 days. This coincides with the introduction of Stage 3 ‘Stay at Home’ coronavirus restrictions across metropolitan Melbourne and the Mitchell Shire.

The first tweet containing the #DictatorDan hashtag was authored on 3 April 2020 by an anonymous fringe account (@CCPIsWatching, 2020), but received no engagement. The tweet has since been deleted. The anti-Chinese stance implied by the handle of this account – and also demonstrated by its ‘real’ name, ‘CCPVIRUS’, which echoes US President Donald Trump’s Sinophobic description of COVID-19 as the ‘China virus’ – is unlikely to be an accident: #DanLiedPeopleDied can be regarded as a memetic variation on the globally circulating #ChinaLiedPeopleDied hashtag that, along with racist hashtags such as #ChinaVirus and #KungFlu, has contributed to a rise in Sinophobia and broader anti-Asian racism (Timberg and Chiu, 2020).

From 1 March to 16 May the #DictatorDan hashtag was only used 282 times, with a total of 92 retweets and 427 likes during that period. Possibly prompted by such low-level circulation, it was the Liberal state MP Tim Smith who arguably set off the viral dynamics of #DictatorDan on Twitter. On 17 May, he created a Twitter poll asking whether to label Dan Andrews ‘Dictator Dan’ or ‘Chairman Dan’ (@TimSmithMP, 2020; Figure 1). This tweet was preceded by several weeks of public name-calling by Smith and another MP, Bernie Finn, who on 9 May called Andrews ‘Kim Jong Dan’ and ‘Despot Dan’ in a Facebook post (Bernie Finn, 2020). Notably, both Smith and Finn were criticised by Victorian opposition leader Michael O’Brien (Ilanbey, 2020). Nonetheless, Smith’s Twitter poll on 17 May attracted considerable engagement and generated a substantial increase in #DictatorDan tweets (857 that day, or three times the total previous activity).

Like the #DanLiedPeopleDied hashtag, the ‘Chairman Dan’ label also carried Sinophobic associations, referencing Chairman Mao. The image of Premier Andrews in Mao’s famous olive green cap, trousers and button-up shirt later became a frequent trope in the editorial cartoons of News Corporation papers (e.g. The Mocker, 2020).

After this initial spike of #DictatorDan tweets, @TimSmithMP posted no further tweets containing the hashtag, and the overall hashtag volume subsided for some time. Nevertheless, Smith’s concerted efforts to push the ‘Dictator Dan’ nickname – and his success with the viral Twitter poll that gained social and news media attention – effectively established the epithet and encouraged fringe accounts to continue the campaign. Partisan news media such as the tabloid *Herald Sun* and commentators on TV channel *Sky News* also heavily pushed the ‘Dictator Dan’ narrative in their reporting, attacking Andrews’s handling of the Victorian outbreak.

**Figure 4.**
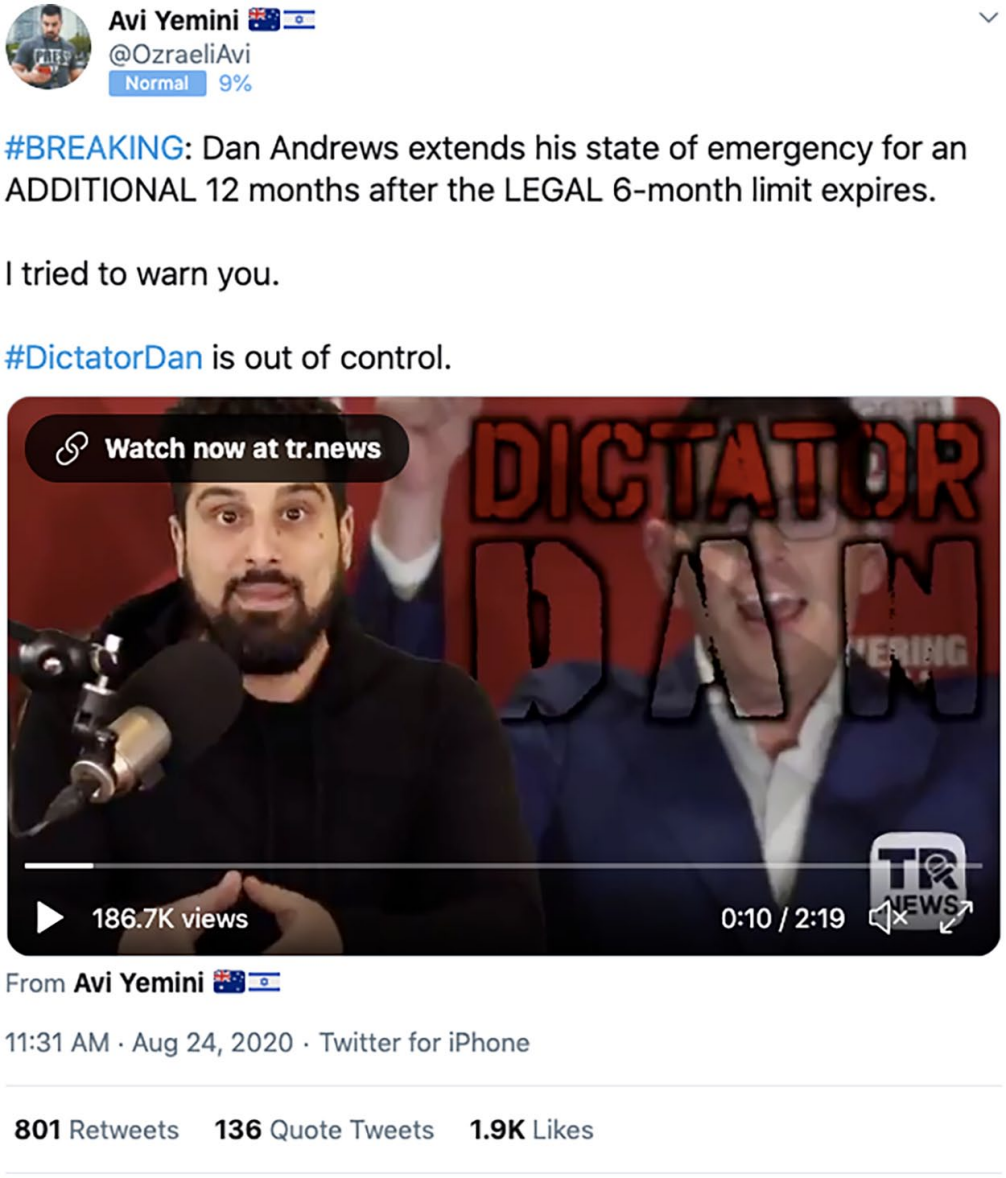
#DictatorDan ‘news report’ tweet by Avi Yemini (@OzraeliAvi, 2020b).

Following this initial burst of activity, however, the activities that contributed the most to subsequent growth in hashtag activity were tweets and articles by far-right commentator Avi Yemini, who describes himself as a journalist reporting for fringe outlets *TR News* and *Rebel News*, and by a loosely coordinated group of highly active fringe accounts. On 24 August, *Sky News* published a widely circulated story, ‘Andrews wants to “remain as Dictator Dan” for another 12 months’ (Sky News, 2020), and this coincided with a large spike in #DictatorDan tweeting on that day. But *Sky News* and other media reporting were not at the centre of this new activity: instead, a ‘video report’ by Yemini (Figure 4) was heavily amplified by his followers and the broader community of hyper-partisan accounts that form the core interest group for fringe, far-right politics in the Australian Twittersphere. The 24 August spike for #DictatorDan thus consists mainly of retweets of Yemini’s posts (2279 out of 5823 tweets that day, or 39%), but contains no tweets of *Sky News* URLs.

Likewise, Yemini and a core of highly active fringe accounts played an important role in the dynamics of #DanLiedPeopleDied. Yemini attracted 1 in 10 of all retweets for this hashtag, and over a third of all #DanLiedPeopleDied retweets (38%, or 4504 tweets) were retweets of only 10 unique accounts, including Yemini. Originally, #DanLiedPeopleDied had seen very little activity: 14 of the 44 #DanLiedPeopleDied tweets posted between 1 March and 10 August were authored by the anonymous account @Anti_ANTIFA2, now suspended by Twitter (as of 1 October 2020). The hashtag was further circulated at low volume by a group of other fringe, hyper-partisan accounts, until one of these accounts managed to generate a greater level of engagement with a tweet in the afternoon of 11 August, receiving 101 retweets that day (@bueller_tom, 2020a).

On the morning of the following day, the same account spearheaded an orchestrated campaign to push the hashtag onto Twitter’s Australian trending topics list (@bueller_tom, 2020b). This in turn attracted the attention of Yemini, who that same day posted seven original tweets and seven retweets containing #DanLiedPeopleDied to his 128,000 followers. An explosion of activity around this hashtag ensued; of the 6078 tweets posted on 12 August, 994 (16%) were retweets of Yemini’s posts. This sudden increase in posts to the hashtag also coincides with Victorian Opposition Leader Michael O’Brien’s claim that the Andrews government had ‘lied to parliament and lied to Victorians’ (Piovesan, 2020) – though we note that there is no evidence that O’Brien’s rhetoric was influenced by the hashtag’s prominence in Twitter’s trending topics that day.

**Figure 5.**
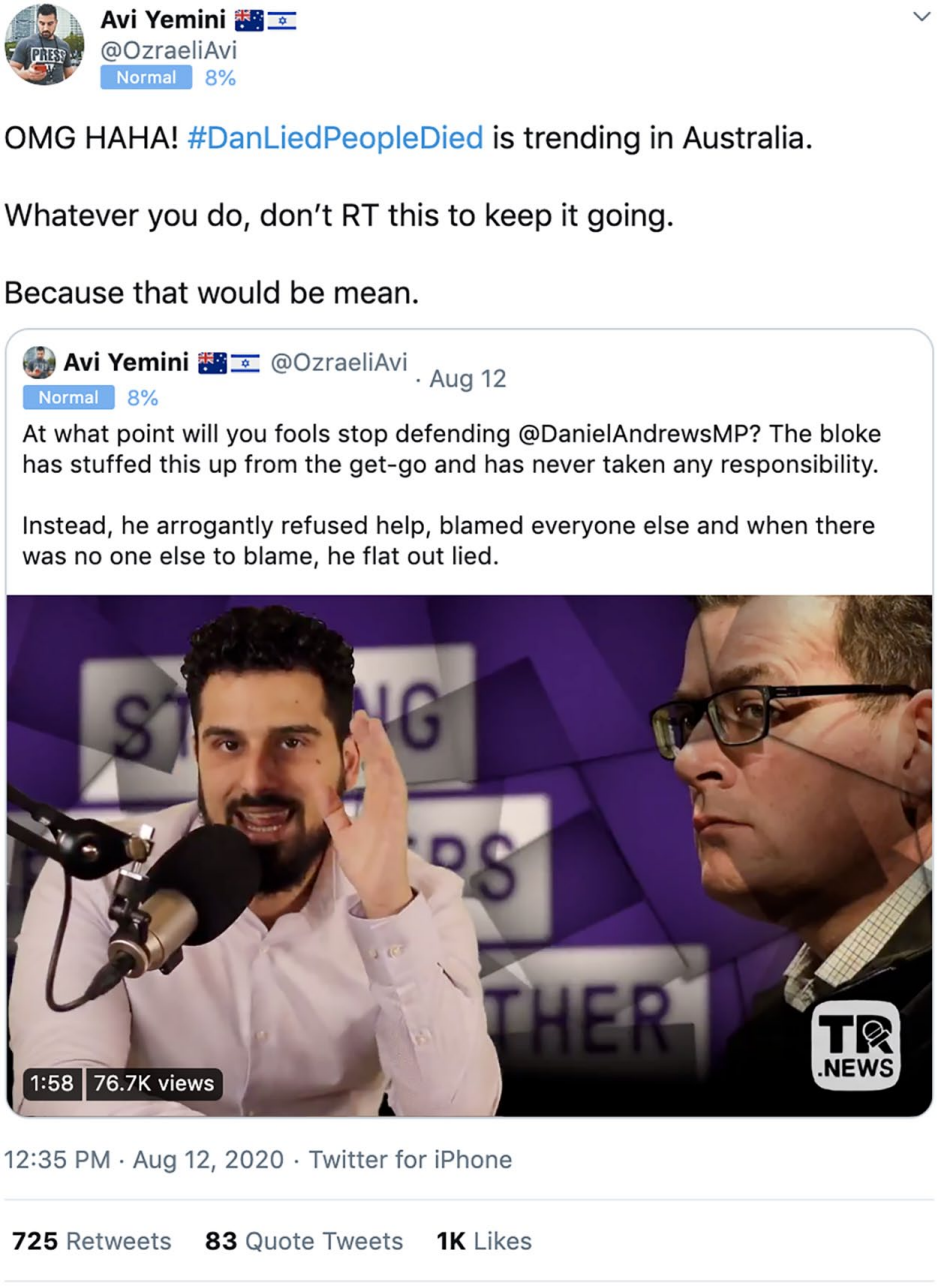
Tweet by Avi Yemini calling for a coordinated action to keep #DanLiedPeopleDied trending on Twitter (@OzraeliAvi, 2020a).

Figure 5 shows one of Yemini’s viral tweets on 12 August, calling for a coordinated campaign to keep #DanLiedPeopleDied trending. This represents the practice of ‘brigading’ (Massanari, 2017), where a group of accounts exploit platform features (such as the vote button on Reddit or the retweet button on Twitter) to engage in the coordinated amplification (e.g. retweeting) or suppression (e.g. downvoting) of particular content and/or individuals.

Yemini has been banned repeatedly from Facebook for engaging in hate speech, and has attracted controversy because of his extremist views and criminal history (Wilson, 2020); his tweets similarly show a degree of toxic behaviour. Although his tweet *prima facie* seems to be asking followers *not* to retweet the hashtag #DanLiedPeopleDied ‘because that would be mean’, implicitly it encourages them to do just that. Ultimately, this orchestrated campaign by loosely coordinated far-right fringe accounts, amplified and endorsed by Yemini’s own, considerably more influential account, was successful: the spike on 12 August effectively ‘launched’ #DanLiedPeopleDied on Twitter, after which it sustained engagement.

### Most prominent accounts

Examining these dynamics further, we turn to the activity patterns within the two anti-Dan hashtags. The most prominent accounts in these hashtags – that is, those accounts that received the greatest number of @mentions and retweets – are listed in Table 1, along with their own tweet activity and a classification of their account types. As the table shows, the activities within #DictatorDan and #DanLiedPeopleDied primarily address elected state and federal politicians, state police, news media and journalists, most of whom are tweeted at but do not themselves actively use these hashtags; as a result, the hashtagged tweets received by such accounts are exclusively @mentions rather than retweets. The accounts that do substantially contribute to the hashtag *and* receive a significant amount of @mentions and retweets belong exclusively to fringe hyper-partisans, and indeed the top 20 most retweeted accounts in these hashtags receive some 47% of all retweets containing these hashtags. Avi Yemini is particularly central as an opinion leader for this network: he receives the largest number of retweets by some margin (15% of all retweets), and only Premier Andrews, Victoria Police and Prime Minister Morrison are @mentioned in a greater number of hashtagged tweets.

**Table 1.** Top 20 most mentioned accounts in the anti-Dan hashtags.

Account	Mentions received	Retweets received	Total interactions received	Tweets to hashtags	Account type
danielandrewsmp	13,622	0	13,622	0	Politician
victoriapolice	4405	0	4405	0	Public authority
scottmorrisonmp	2718	0	2718	0	Politician
ozraeliavi	2116	11,891	14,007	81	Fringe hyper-partisan
ausantileft	1990	2351	4341	950	Fringe hyper-partisan
skynewsaust	1649	0	1649	0	News media
theheraldsun	1630	0	1630	0	News media
timsmithmp	1493	0	1493	0	Politician
michaelobrienmp	1028	0	1028	0	Politician
aussieval10	1016	4998	6014	285	Fringe hyper-partisan
7newsmelbourne	950	0	950	0	News media
victoriancho	908	0	908	0	Public authority
theage	890	0	890	0	News media
9newsmelb	876	0	876	0	News media
polibard	781	0	781	0	Fringe hyper-partisan
victorianlabor	767	0	767	0	Political party
jeff_kennett	757	0	757	0	Politician (former)
ritapanahi	702	0	702	0	Journalist
fionapattenmlc	689	0	689	1	Politician
riseupmelbourne	633	19	652	1	Fringe hyper-partisan

In fact, focussing exclusively on the second major spike of #DictatorDan tweets on 6 September 2020, we find that fully one-fifth are retweets of Yemini’s tweets (1974 out of 9187 tweets), alongside fringe accounts @aussieval10 and @ausantileft who together receive another 10% of the total retweets (618 and 315, respectively). This is in spite of the fact that Yemini had only posted a total of 10 tweets to the #DictatorDan hashtag that day – yet, his large base of 128,000 followers positioned him as an opinion leader for the hashtag.

These and other fringe actors also sought the attention of elite actors in the public sphere by directly engaging them through @mentions. Unsurprisingly, as the target of the #DictatorDan hashtag, @DanielAndrewsMP receives the most @mentions that day (802); these are overwhelmingly negative, even to the point of wishing imprisonment and death for him and his team. Tweets also @mention conservative politicians such as former Premier Jeff Kennett, MP Tim Smith, Opposition Leader Michael O’Brien, as well as various news outlets and journalists.

### The origin and dynamics of the #IStandWithDan hashtag

The first #IStandWithDan tweet in support of Premier Andrews was posted on 22 March 2020, but received little engagement. It was followed in subsequent days by a trickle of tweets by ordinary accounts showing their support for Dan Andrews, and growing calls for coordinated action to make the hashtag trend. The hashtag went viral on 8 July 2020 with nearly 1600 tweets: Stage 3 ‘Stay at Home’ restrictions came into effect across metropolitan Melbourne and the Mitchell Shire that day at 23:59, and the spike in activity is clearly in response to this event; indeed, the following day – the first under Stage 3 – saw the greatest activity for this hashtag, with 14,534 tweets.

The next substantial peaks in activity occurred on 2 August (8689 tweets), 12 August (9340) and 6 September (12,007). The 2 August peak coincided with the ‘Stage 4’ lockdown announcement; the 12 August peak responded to the largest daily volume in #DanLiedPeopleDied tweets, and media attention to that hashtag; the 6 September peak followed the 5 September ‘Freedom Day’ protest (The Age, 2020) and coincides with the largest spike in #DictatorDan tweets (9187), as Andrews came under coordinated attack from conservative media outlets.

Activity for this hashtag thus generally appears to respond to the stages of lockdown in Victoria, to coordinated attacks on Dan Andrews from conservative media, which heavily amplified ‘Liar Dan’ and ‘Dictator Dan’ tropes across multiple news outlets on key days, and to the social media campaigns of Twitter activists as we have seen them in our analysis of the #DictatorDan and #DanLiedPeopleDied hashtags. In the pro-Dan hashtag, activity is similarly concentrated around a central core of participants; to demonstrate this, we apply the well-known 1/9/90 rule (Bruns and Stieglitz, 2012; Tedjamulia et al., 2005) to divide the total number of participants in each hashtag into the top 1% of most active contributors, the next 2%–9% of frequently active contributors, and the bottom 90% of least active contributors, and similarly divide the total number of all retweet recipients in each hashtag into the top 1% of most retweeted accounts, the 2%–9% of frequently retweeted accounts, and the bottom 90% of least retweeted accounts. For each of these groups, we then calculate their total share of all the tweets or retweets posted to their hashtag, respectively (Table 2).

**Table 2.** Contribution of participant percentiles to total tweet/retweet count per hashtag.

	#DictatorDan	#DanLiedPeopleDied	#IStandWithDan
Tweets sent			
Top 1% of contributors	36,882 (34%)	5396 (26%)	89,114 (32%)
2%–10% of contributors	40,553 (38%)	7434 (36%)	114,686 (42%)
Bottom 90% of contributors	30,409 (28%)	7963 (38%)	71,773 (26%)
Total	107,784 (100%)	20,793 (100%)	275,573 (100%)
Retweets received			
Top 1% of recipients	35,702 (52%)	4107 (35%)	91,073 (46%)
2%–10% of recipients	21,166 (31%)	4804 (41%)	80,643 (41%)
Bottom 90% of recipients	11,826 (17%)	2912 (25%)	25,490 (13%)
Total	68,694 (100%)	11,823 (100%)	197,206 (100%)

This analysis shows that, with respect to active contributions, #DictatorDan and #IStandWithDan are similarly concentrated around a hard core of participants: the top 1% of most active accounts posted some one-third of all tweets with these hashtags (34% for #DictatorDan and 32% for #IStandWithDan), while the top 1% of participants in #DanLiedPeopleDied contributed only just over one quarter (26%). Taking the two most active groups together, in fact, #IStandWithDan even turns out to be slightly more concentrated around its core: the top 10% of participants posted some 74% of all its tweets, compared to 72% for #DictatorDan and 62% for #DanLiedPeopleDied.

The same patterns apply also to the retweeting behaviour: 52% of all #DictatorDan retweets, and 46% of all #IStandWithDan retweets, amplify the posts of their top 1% of retweet recipients, compared to only 35% for #DanLiedPeopleDied; taking the two most frequently retweeted groups together, 87% of all retweets in #IStandWithDan and 83% of all retweets in #DictatorDan provide amplification for the top 10% most prominent accounts, compared to 76% for #DanLiedPeopleDied. All three hashtags, but especially the two considerably larger hashtags #DictatorDan and #IStandWithDan, are thus highly effective vehicles for providing amplification and channelling attention towards a comparatively small, highly active and highly visible subset of all participants.

At first glance, the actors in the top 20 of most central accounts for #IStandWithDan, shown in Table 3, also appear similar to those for the anti-Dan hashtags (Table 1); they represent a mix of politicians, news media, journalists and fringe hyper-partisan accounts. However, the interaction dynamics are fundamentally different. First, while the @mentions of Dan Andrews in the anti-Dan network were predominantly negative and critical, @mentions of his account in the #IStandWithDan network are of course overwhelmingly positive and supportive; additionally, they also represent a much greater absolute volume of interactions with Andrews’s account (51,292 @mentions and retweets, as compared to 12,459 for the anti-Dan hashtags). This difference in sentiment is stark, but – given the explicit sentiment of these hashtags – hardly surprising.

**Table 3.** Top 20 most mentioned accounts in the pro-Dan hashtag.

Account	Mentions received	Retweets received	Total interactions received	Tweets to hashtags	Account type
danielandrewsmp	61,207	0	61,207	0	Politician
timsmithmp	5451	0	5451	0	Politician
scottmorrisonmp	5098	0	5098	0	Politician
Abcnews	4035	0	4035	0	News media
melbourne_says	3869	5739	9608	167	Fringe hyper-partisan
cathlandrews	3314	3170	6484	5	Public figure
skynewsaust	2707	0	2707	0	News media
theheraldsun	2500	0	2500	0	News media
sophieelsworth	2360	460	2820	7	Journalist
lesstonehouse	2081	1048	3129	107	Fringe hyper-partisan
theage	1949	0	1949	0	News media
david_speers	1920	0	1920	0	Journalist
alexdevantier	1883	3497	5380	517	Politician
breakfastnews	1634	0	1634	0	News media
michaelobrienmp	1613	0	1613	0	Politician
joshfrydenberg	1582	0	1582	0	Politician
rachelbaxendale	1543	0	1543	0	Journalist
australian	1535	0	1535	0	News media
belindajones68	1479	4910	6389	156	Fringe hyper-partisan
jeff_kennett	1475	0	1475	0	Politician (former)

**Figure 6.**
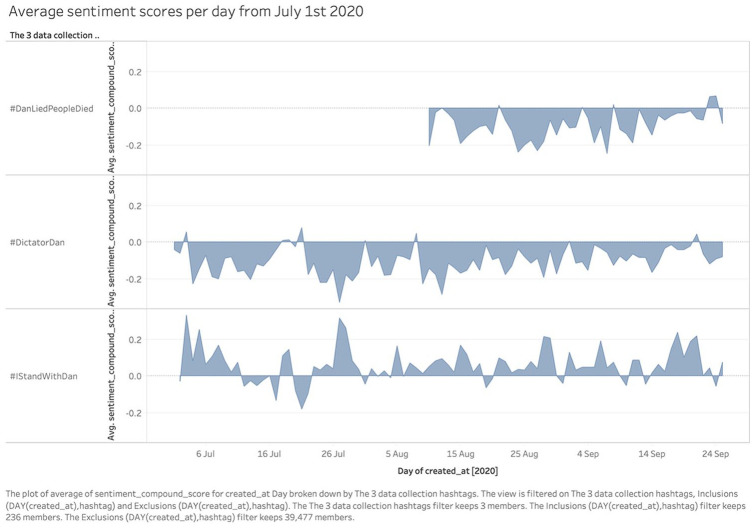
Average compound sentiment score per day for each hashtag, from 1 July 2020.

Using the compound sentiment score produced by the VADER algorithm, Figure 6 shows the average sentiment score per day for each hashtag, from –1 (extremely negative) to +1 (extremely positive). In line with the distinct dynamics of each Twitter hashtag, the emotional valence of #IStandWithDan was largely positive throughout its timeline, whereas the anti-Dan hashtags were overwhelmingly negative; indeed, #DictatorDan and #IStandWithDan are at times moving in parallel with each other, but with opposite emotional valence. This reveals the amount of polarisation between the communities of participants in these opposing Twitter hashtags, and highlights the important role of emotion in the dynamics of such discussions about Premier Andrews and the Victorian lockdown measures.

Second, although the accounts of conservative news media such as *Sky News* and *The Herald Sun* are prominent in the #IStandWithDan interactions network (as they are in the anti-Dan network), the goal of #IStandWithDan tweeters was not to get the attention of these news outlets in order to seek amplification; rather, participants used the hashtag to criticise their coverage of the Victorian lockdown, and to reshape and control the narrative by replying to their tweets *en masse*. Many such tweets attacked conservative news outlets for their persistent anti-Andrews reporting, pointed out the apparent coordination of critical coverage across outlets operated by the NewsCorp stable, or highlighted the conflicting rhetoric used by particular journalists, columnists and pundits. By contrast, the news outlets whose content is actually shared in #IStandWithDan tweets predominantly include the American news site *CNN*, the Australian public service broadcasters *ABC News* and *SBS News*, and the progressive outlet *The New Daily*.

Third, journalists are therefore central in the #IStandWithDan network not because fringe accounts are seeking to get their attention in the hope that they will boost their own messages (as is the case in the anti-Dan network), but instead, because participants address these journalists’ accounts to voice criticism – especially towards @sophieelsworth (*Herald Sun*), @rachelbaxendale (*The Australian*) and @DavidSpeers (*ABC News*). This criticism centrally addresses the perceived bias of these journalists in reporting on the Victorian lockdown, and particularly in questions directed at Premier Andrews during his daily press conferences and in other appearances. Sadly, especially in tweets directed at the female journalists, we also observe a certain degree of problematic, abusive content.

### Account characteristics for the pro- and anti-Dan hashtags

Table 4 provides summary statistics from a qualitative content analysis of the top 50 accounts (by tweet frequency) posting to each hashtag, a group we describe as high-frequency accounts. By applying the manual coding scheme outlined in the ‘Methods’ section, we identify a considerable proportion of these accounts as anonymous sockpuppets – that is, as accounts with incomplete or fabricated profile details: by our definition, over half of the high-frequency accounts posting to the anti-Dan hashtags (54%) qualify as sockpuppets, compared to one-third (34%) of the high-frequency accounts posting to #IStandWithDan. Notably, at the time of writing in October 2020, three of the 50 high-frequency accounts from the anti-Dan hashtags had already been suspended by Twitter, while none from #IStandWithDan had been suspended. Furthermore, the high-frequency sockpuppet accounts from the anti-Dan hashtags posted 14% and 9% of the total tweets in #DanLiedPeopleDied and #DictatorDan, respectively. This is higher than the proportion for #IStandWithDan, where 6% of tweets were sent by high-frequency sockpuppet accounts.

**Table 4.** Activity of high-frequency accounts posting to each hashtag.

Hashtag	Number of authentic accounts (% of top 50 accounts)	Number of sockpuppet accounts (% of top 50 accounts)	Number of suspended accounts (% of top 50 accounts)	Number of tweets sent (% of all tweets in hashtag)	Number of tweets by sockpuppet and suspended accounts (% of all tweets in hashtag)
#IStandWithDan	33 (66%)	17 (34%)	0	39,046 (14%)	15,608 (6%)
#DanLiedPeopleDied	21 (42%)	27 (54%)	2 (4%)	5073 (24%)	2838 (14%)
#DictatorDan	22 (44%)	27 (54%)	1 (2%)	19,335 (18%)	10,226 (9%)

**Figure 7.**
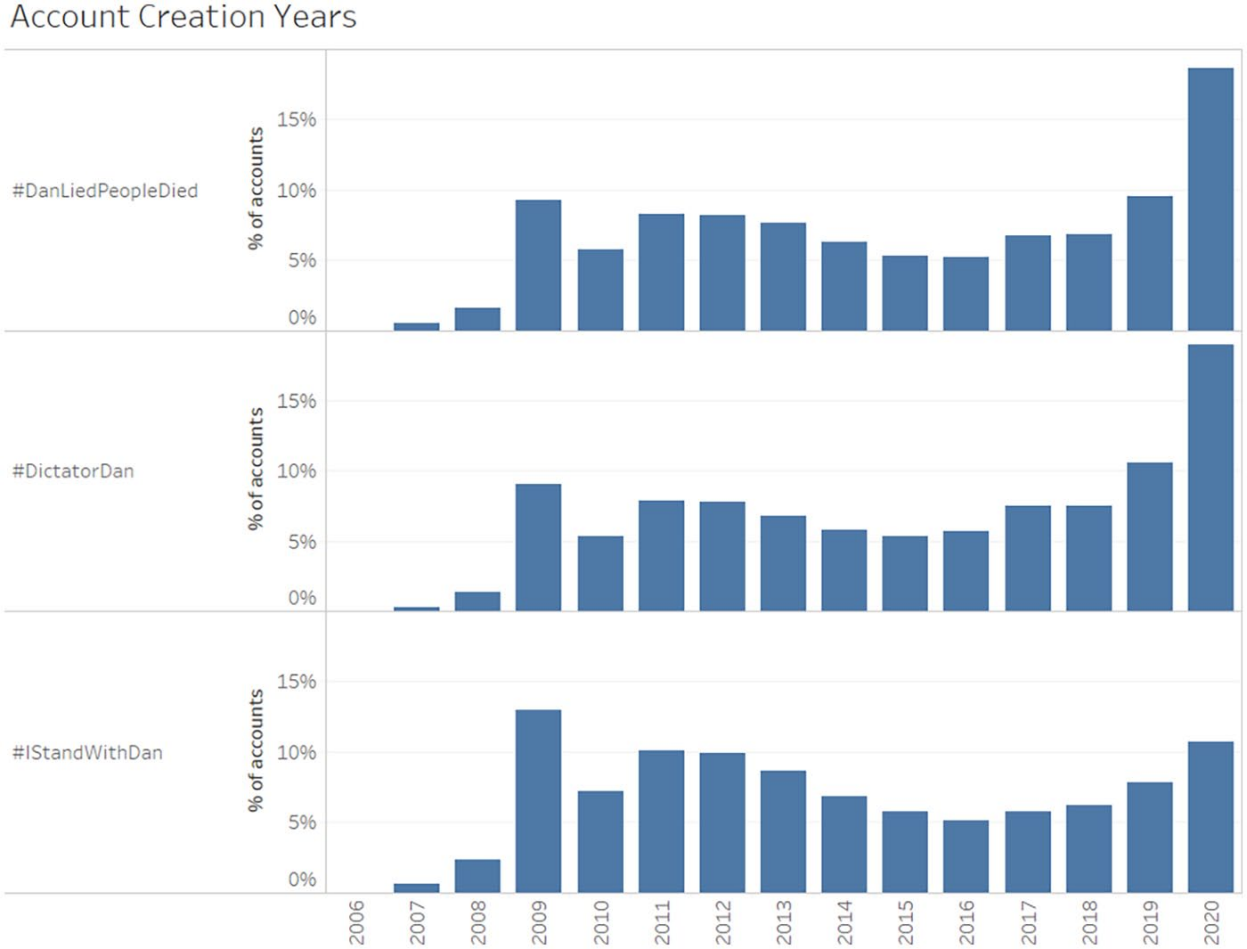
Distribution of account creation years per hashtag.

We also find that many of the accounts participating in anti-Dan hashtags were created more recently than those engaging in pro-Dan hashtags. Figure 7 shows that nearly one-fifth of all accounts participating in #DanLiedPeopleDied (18.6%, or 1036 accounts) and #DictatorDan (19%, or 3432 accounts) were created in the year 2020, compared to just over one-tenth for #IStandWithDan (10.7%, or 2924 accounts). Indeed, more than 6% of the accounts participating in either anti-Dan hashtag were created just in the 3-month period since 1 July 2020 – that is, from the time just prior to the Stage 3 ‘Stay at Home’ restrictions coming into effect, and coinciding with a substantial increase in Twitter activity across all hashtags (Figure 3): 6.2% of #DictatorDan accounts were created in July to September 2020, and 6% for #DanLiedPeopleDied. This is double the 3.1% of #IStandWithDan accounts created during that time.

It is conceivable, of course, that the lockdowns, the heated discussion surrounding the lockdowns and other public health measures, and the overall pandemic crisis would have resulted in an influx of new users to Twitter, during 2020 overall and since the introduction of stricter lockdown measures in particular; previous studies have documented similar spikes in new account sign-ups in the context of other crisis events such as the Queensland floods, Christchurch earthquakes, or Sendai tsunami in 2011 (Bruns et al., 2014). However, it appears highly unlikely that this influx would have occurred in such significantly uneven patterns, resulting in a proportionally greater take-up of Twitter by the opponents rather than supporters of the Andrews government.

A more likely explanation, and one also in keeping with our observations of the greater percentage of fabricated sockpuppet profiles among the most active accounts in the anti-Dan hashtags, is that the fringe activists promoting the #DictatorDan and #DanLiedPeopleDied hashtags have engaged in the deliberate creation of new, ‘fake’ accounts that are designed to generate the impression of greater popular support for their political agenda than actually exists in the Victorian population (or at least in its representation on Twitter), and to use these fabricated accounts to fool Twitter’s trending topic algorithms into giving their hashtags greater visibility on the platform. By contrast, the general absence of such practices means that #IStandWithDan activity is a more authentic expression of Twitter users’ sentiment.

We also note here that this use of ‘fake’ accounts to artificially boost the visibility of topical hashtags is distinct from more blatant uses of entirely automated accounts, usually described as bots. Using the Botometer tool and setting a threshold of 0.9 for its Completely Automated Probability (CAP) score – ‘the probability, according to our models, that an account with this score or greater is controlled by software, i.e., *is* a bot’ (Botometer, 2020, emphasis original) – we detect only a vanishingly small number of likely bots across our samples of the 1000 most active accounts in each of the three hashtags (Table 5).

**Table 5.** Results of bot analysis and bot engagement for each hashtag.

Hashtag	Number of likely bot accounts (CAP > 0.9) in the top 1000 most active accounts	Total tweets sent	Total retweets received	Total likes received
#DanLiedPeopleDied	15	143	27	57
#DictatorDan	25	121	29	56
#IStandWithDan	11	44	2	7

CAP: completely automated probability.

At 15 and 25 bots, respectively, the anti-Dan hashtags feature nearly four times the number of bot accounts compared to #IStandWithDan’s 11 bots, but such numbers are very low in the context of the thousands and tens of thousands of unique accounts posting to these hashtags. In addition, bot accounts were not particularly active: #DanLiedPeopleDied bots sent 11 tweets on average, followed by five tweets on average for #DictatorDan and four tweets on average for #IStandWithDan. Engagement with bot-like accounts, as measured by the total number of retweets and likes they received, was considerably higher for the anti-Dan hashtags, yet overall engagement was low and therefore the impact of bot-like accounts in terms of reach is minimal.

These patterns remain even if we extend our analysis to include less obviously bot-like accounts, as assessed by Botometer’s CAP score. Turning attention to the *distribution* of bot probabilities, we observe a higher probability of bot-like activity for the anti-Dan hashtags as compared to #IStandWithDan. The mean CAP score for #DanLiedPeopleDied is 0.6, and that for #DictatorDan is 0.63, while #IStandWithDan sees a mean of 0.51. A two-sided independent t-test confirms that the differences in mean CAP scores between the anti-Dan hashtags and #IStandWithDan are statistically significant (p < 0.000001); the difference in mean between the two anti-Dan hashtags is also statistically significant (p < 0.01).

These findings suggest that although there are not many *completely automated* accounts (i.e. bots), the accounts engaged in the anti-Dan hashtags present significantly more *like bots* in some of their features. As Botometer scores operationalise some of the features also used in our manual coding for sockpuppet accounts (such as incomplete or fabricated profile information), and also take into account excessive tweeting and retweeting activity, the higher probability scores for accounts in the anti-Dan hashtags is likely to reflect the almost bot-like artificial and inauthentic amplification activities that these accounts are engaged in, even if they remain human-controlled or at best ‘hybrid’ accounts (controlled by humans but utilising automation techniques such as tweet scheduling or automated retweeting). This difference in overall Botometer ratings thus supports and validates the results of our manual coding of the most active accounts in each hashtag for their sockpuppet features.

## Discussion and conclusion

Although all three hashtags respond to the same issues and engage with many of the same actors, then, the dynamics of their information flows differ in important ways. In all three hashtags, the tagging of elite actors through @mentions in tweets can be seen as attempting to initiate a process of ‘reverse agenda setting’ (Russell Neuman et al., 2014; Towner and Muñoz, 2018), where participants on the periphery seek to gain visibility for their views by seeking amplification from elite actors. However, for the case of #DanLiedPeopleDied and #DictatorDan, this does not fully capture the multi-directional diffusion dynamics and interaction structures. Rather, they represent what Ognyanova (2017) describes as a ‘Type II’ multi-step flow network model.

Where the original two-step flow model envisaged mass media as influencing local opinion leaders, who would in turn influence the opinions of the communities surrounding them, an extension of this model to a multi-step flow model initially simply anticipated the further dissemination of views and opinions between more or less connected members of those communities, offline as well as online. Ognyanova describes this as ‘Type I’ of the multi-step flow model: a model which retains the top-down primacy of the mass media as a source of ideas that then simply circulate more extensively among local communities.

By contrast, the network-based ‘Type II’ of the multi-step flow model reduces this primacy and places greater emphasis on what Habermas (2006) has described as the ‘wild flow of messages’ (p. 415) among the community. Here, mass media do not occupy a privileged position outside the social structure of the community, but instead are embedded within it. In the case of the anti-Dan hashtags, news media are not setting the agenda in a top-down fashion (i.e. producing news with which the public engage), but, along with potentially sympathetic journalists and politicians, are *addressed strategically* by highly active hyper-partisan opinion leaders and their followers in order to facilitate the further dissemination of opinions and rhetoric that are critical of Premier Andrews. Such actions are not focussed exclusively on generating greater take-up of these views on Twitter alone, then; rather, by targeting politicians and journalists the proponents of these views are attempting to transport them into other media forms as well.

But in these anti-Dan hashtags, this multi-step flow relationship is complex and recursive, and not simply reducible to direct or reverse agenda-setting. The ‘Liar Dan’ narrative embraced by some conservative news media outlets is qualitatively distinct from #DanLiedPeopleDied, which as noted is also a variation on the Sinophobic #ChinaLiedPeopleDied hashtag. While anti-Dan Twitter activists were promoting similar narratives to those pursued by partisan news media, they made them their own through their social meaning-making and online content production, and relied heavily on meme warfare and pre-existing racist discourses in attracting online engagement and in pushing their agendas.

Although some conservative news outlets also repeatedly framed the Victorian lockdown using the ‘Liar Dan’ and ‘Dictator Dan’ narratives, the peaks in activity for these hashtags on Twitter – and subsequent sustained levels of increased activity – were primarily driven by the concerted efforts of these right-wing ‘clicktivists’ (Freelon et al., 2020) and their leaders. Anti-Dan hashtag tweets do frequently cite critical news coverage in support of their own perspectives, however: links embedded in these tweets predominantly pointed to the mainstream news site *news.com.au*, the Melbourne broadsheet *The Age*, conservative TV channel *Sky News*, Canadian far-right outlet *Rebel News* (for which Yemini serves as Australian bureau chief), conservative national broadsheet *The Australian*, mainstream TV bulletin *Seven News*, and the staunchly conservative political magazine *The Spectator*.

Overall, then, the flow patterns we observe with the anti-Dan hashtags should more properly be described as follows:

- An undercurrent of antipathy towards the pandemic lockdown measures circulates on Twitter;
- Mainstream and especially conservative news media cover the actions of the Victorian state government from a critical perspective;
- Some such reporting is used by anti-Andrews activists on Twitter to sharpen their attacks against Andrews (see, for example, the Yemini tweet shown in Figure 4), but in doing so, they also draw on pre-existing memes and rhetoric from other sources (including the Sinophobic #ChinaLiedPeopleDied), and adapt these to the local situation;
- Such rhetoric is circulated by ordinary users and their hyper-partisan opinion leaders on Twitter, amplified by spam-like tweeting behaviours and purpose-created sockpuppet accounts, and aggregated by using anti-Dan hashtags such as #DictatorDan and #DanLiedPeopleDied as a rallying point;
- This content is in turn directed at news media, journalists, and politicians (as Table 1 shows) in the hope that it may find sympathy and endorsement, in the form of retweets on Twitter itself or take-up in their own activities outside of the platform (including MP Tim Smith’s Twitter poll, in Figure 1);
- And such take-up in turn encourages further engagement in anti-Dan hashtags on Twitter, repeatedly also pushing them into the Australian trending topics list.

This linear depiction is necessarily a simplification of such multi-step flows, of course; in reality, many of these stages are happening simultaneously for the specific messages and memes produced by activists, and the overall process represents a feedback loop that continuously seeks to reinforce and amplify its messages.

In contrast, the #IStandWithDan hashtag is governed by rather different dynamics. Whereas the anti-Dan hashtags are involved in a networked multi-step flow process that involves conservative media, mainstream politicians, fringe opinion leaders and a loosely coordinated community of hyper-partisan accounts, and operates as a feedback loop that perpetuates aggressive rhetoric critical of the Andrews government and its pandemic control measures, #IStandWithDan appears considerably more clearly as an ad hoc public (Bruns and Burgess, 2015) engaging in a form of hashtag activism (Jackson et al., 2020) that simultaneously shows support for Premier Andrews and criticises perceived bias from allegedly partisan media and journalists. Yet, there are few attempts to enrol potentially sympathetic politicians, journalists and media outlets in the pro-Andrews campaign, nor is there evidence of a concerted effort to utilise newly created sockpuppet accounts in artificially amplifying its views; this is also simply unnecessary because the number of accounts and volume of tweets contributing to #IStandWithDan organically is already larger than those for the anti-Andrews hashtags.

Thus, #IStandWithDan is an example of broadly left-wing ‘clicktivism’ (Freelon et al., 2020), where a large number of ordinary Twitter accounts on the periphery of the public sphere utilise the affordances of social media to show their support for a particular political cause and engage in critical discourse. By contrast, the anti-Dan hashtags can be regarded as a form of right-wing ‘clicktivism’ – but, in line with the findings of Freelon et al. (2020), the right-wing activists strategically work *with* sympathetic media and politicians to spread their messages: both in trying to attract their attention and amplification in order to influence public debate, and in responding to (if not directly amplifying) the narratives and agendas of these media outlets, particularly when they engage in coordinated media attacks on the Andrews government. This is not the case for the left-wing activists, who primarily engage with news media to criticise their coverage. Thus, even though the pro- and anti-Dan activity can both broadly be described as hashtag clicktivism, these publics follow thoroughly different logics.

The hashtag campaigns we have examined here demonstrate, on both sides of politics, a sophisticated understanding of Twitter and its potential for the mobilisation of supporters; furthermore, especially the hyper-partisan campaigners opposing the Victorian government’s lockdown measures also exhibit a highly developed sense of the strategies required for making their minority views more visible to the general Twitter public on one hand, and to the news outlets, journalists and politicians who might be persuaded to transport them to the general Victorian and Australian public on the other hand. The success of well-known far-right commentators in pursuing such strategies is especially problematic, and indicates the vulnerability of Australian mainstream politics and media to actors who hide extremist politics under a mediagenic veneer (but we note that this is not necessarily a problem limited to the far right; in other contexts, far-left actors may have been just as successful in employing such strategies).

Especially where such actors employ coordinated inauthentic behaviours, such as the creation and use of sockpuppet accounts, to make their views appear more popular than they are, mixed-methods approaches as we have employed them here are crucial for detecting such manipulation. Our study thus also points to an urgent need for journalistic and political stakeholders to enhance their own social media literacies in order to avoid falling prey to such deliberate manipulation.

In this way, the findings of this study highlight the continuing problem of the ‘oxygen of amplification’ whereby journalistic amplification of false or misleading content carries both costs and benefits (Phillips, 2018). In particular, Phillips (2018) observes that ‘amplification makes particular stories, communities, and bad actors bigger – more visible, more influential – than they would have been otherwise’ (p. 4). The Victorian lockdown was a divisive political issue in Australia, but the risk for journalistic coverage of the polarised activity on social media is that it simply fuels the issue and creates further polarisation, in turn benefitting manipulators and encouraging them to keep going, learn and adapt their strategies. Conversely, *not* amplifying such news stories carries various risks such as missed opportunities to educate the public or other newsrooms getting to the story first and gaining the attention and revenue.

We therefore implore journalists and political stakeholders to adopt expert-informed frameworks for identifying and combatting mis- and disinformation and other problematic content. As a practical recommendation, the ‘Journalism, Fake News and Disinformation handbook’ developed by Ireton and Posetti (2018) provides an excellent resource to gain a critical awareness of, and practical techniques to deal with, the growing problem of ‘information disorder’ in digital media ecosystems (Wardle and Derakhshan, 2017). This study highlights complex and deeply rooted moral questions facing journalists and other stakeholders who have a voice in the public sphere, but also opportunities to positively shape the media ecosystem through increased information literacy and critical awareness of information disorder.

## Notes

1. Full query for Twarc collection: twarc search ‘#dictatordan OR #danliedpeopledied OR #istandwithdan’ \ —profile observatory_prod \ —fullarchive researchdata \ —from_date ‘2020-03-01 00:00:00’ \ —limit 400000 \ —output full_archive_search.jsonl
